# Protecting the vulnerable during COVID-19: Treating and preventing chronic disease disparities

**DOI:** 10.12688/gatesopenres.13181.1

**Published:** 2020-09-09

**Authors:** Linda M. Mobula, David J. Heller, Yvonne Commodore-Mensah, Vanessa Walker Harris, Lisa A. Cooper

**Affiliations:** 1 Department of Medicine, Johns Hopkins School of Medicine, Baltimore, USA; 2 Center for Humanitarian Health, Johns Hopkins Bloomberg School of Public Health, Baltimore, USA; 3 Arnhold Institute for Global Health, Mount Sinai School of Medicine, New York City, USA; 4 Department of Nursing, Johns Hopkins School of Nursing, Baltimore, USA; 5 Center for Health Equity, Johns Hopkins University, Baltimore, USA; 6 Department of Health, Office of the Secretary of Health and Human Resources, Richmond, USA; 7 Department of Health, Behavior and Society, Johns Hopkins Bloomberg School of Public Health, Baltimore, USA

**Keywords:** NCDs, COVID-19, Vulnerable populations

## Abstract

The coronavirus disease 2019 (COVID-19) pandemic has exacerbated health disparities across ethnic and socioeconomic groups. Non-communicable diseases (NCDs) - such as hypertension, diabetes, and obstructive lung diseases – are key drivers of this widening gap, because they disproportionately afflict vulnerable populations.

Vulnerable populations with non-communicable diseases, in turn, are disproportionately affected by COVID-19 itself – but also at increased risk of poor outcomes from those underlying conditions. Proven strategies for NCD control must be adapted to help vulnerable patients react to these dual threats. We detail six key policy interventions – task shifting, workforce protection, telehealth and mobile services, insurance restructuring and increased funding for NCDs, prescription policies for NCDs and community partnerships - to bridge this care gap. Long-term integration of these care models post-COVID-19 may prevent care shocks during future pandemics, bolstering emerging universal primary care models.

## Background

The coronavirus 2019 (COVID-19) pandemic has worsened access to care, disrupted health services, and diverted human resources to the emergency response worldwide. Vulnerable populations have been disproportionately affected: not only people with a low-income such as the internally displaced, but also ethnic minorities such as Black, Indigenous and other People of Color (BIPOC) in the United States and Black and minority ethnic groups (BAME) in the United Kingdom ^1– 3^. COVID-19 has exacerbated the health access disparities these communities already suffer due to the disruptions to service delivery. These groups also disproportionately bear the brunt of selectively-enforced lockdowns and social distancing regulations – especially in settings of food insecurity, limited access to potable water, and scarce jobs ^4– 9^.

Non-communicable diseases (NCDs) - such as hypertension, diabetes, and obstructive lung diseases – are key drivers of these health inequities. Like COVID-19, NCDs disproportionately cluster in low-income and vulnerable populations - and affect these communities more severely and sooner in life, increasing the risk of disease and death from COVID-19 ^10– 14^. The reasons while ultimately biomedical, are the result of economic, social, and cultural factors. Moreover, structural racism has resulted in limited access to resources to meet basic health needs, limited communication channels, and weaker safety nets for communities of color.

These challenges not only increase risk of COVID-19 infection and death among persons with NCDs – they also worsen control of NCDs among vulnerable persons at risk of COVID-19. This disparity is multifaceted: firstly, vulnerable persons with NCDs face a disproportionate burden of the economic, social, and health-related impact of COVID-19 itself, which distracts from NCD self-care. Secondly, they experience the same anxiety associated with fear of contracting the disease as the general population, magnified by concerns about discrimination; and thirdly, they endure worsening of the existing impaired access to NCD care due to social distancing protocols. **To protect vulnerable persons worldwide from the many threats of COVID-19 – including infection, care interruption, and stigma - we must focus on protecting these communities from NCDs.** We must use tried-and-true tactics from the NCD treatment playbook, but revise them: firstly, to focus more on the specific needs of vulnerable populations; and secondly, to adapt to COVID-19 care disruptions ^15^. We propose seven strategies to expressly protect the health of vulnerable populations living with NCDs.

## NCD prevention, control and treatment in vulnerable populations

Government strategies for NCD control in vulnerable populations have long needed to devise low-cost, innovative solutions, due to a combination of massive disease prevalence and among the lowest donor support margins in the already resource-strapped global health community. Fortunately, many of those creative and low-budget strategies have succeeded and can be leveraged to help vulnerable populations to prevent and control NCDs worsened by COVID-19 - despite significant resource gaps.

Disruptions to care have been demonstrated through a World Health Organization (WHO) **rapid assessment survey of service delivery for NCDs during the COVID-19 pandemic.** This survey found that health services have been partially or completely disrupted for hypertension, cancer, and diabetes treatment, as well as diabetes-related complications and cardiovascular emergencies ^15^. Only 66% of all countries and only 61% of LMICs have included continuation of NCD services as part of their pandemic plan.

## Protection and prioritization of healthcare workers

Globally, healthcare workers (HCWs) are at highest risk of COVID-19 infection and mortality. Inadequate access to PPE and poor infection control practices in low resource healthcare settings lead to mistrust from communities, causing underutilization of essential services. Patients may delay or avoid health care altogether because of the perceived threat of COVID-19.

Delivering high quality care for patients with NCDs requires national strategies to minimize infection risk to HCWs and ultimately protect patients from COVID-19. This effort requires protocols specific to low-resource settings to achieve infection protocol in fragile primary care clinics, such as the WHO Infection Prevention and Control protocol for COVID-19.

## Telehealth, M-health and other technologies to improve access to care to manage NCDs

When delivered equitably and effectively, telehealth expands access to NCD care and reduces barriers to care. The evidence is especially robust for home monitoring of patients with NCDs and psychotherapy for behavioral health ^16^. Although most such evidence comes from high income countries, in LMICs, telehealth is an emerging approach to deliver healthcare to manage NCDs. Mobile-health (m-health) management of NCDs in LMICs, where 70% of mobile subscriptions are now found ^17^, is an underutilized strategy - especially in rural areas where in-person care is scarce. One promising approach includes partnerships among internet service providers, health technology companies, and health care providers to develop accessible platforms that enhance communication between patients and frontline workers and improve access to care.

## Shifting and sharing health tasks across the healthcare team

In settings where clinicians are scarce, other providers can assume physicians’ tasks, a strategy called task-shifting. Nurses and volunteers can control blood pressure, treat depression, and educate on tobacco cessation, for instance, as well as doctors ^18^. Telemedicine strategies for non-doctor NCD care - for instance smartphone tracking of blood pressure, or text and telephone correspondence for depression - have emerged over the past 10 years. Furthermore, task-shifting models to treat and prevent COVID-19 have burgeoned over the past few months.

For instance, NCDs peer support groups such as the Chronic Disease Self-Management Program (CDSMP) have already been brought online, and this approach has expanded due to COVID-19 ^19^. The online format not only lowers the logistical and economic cost of participation. It also allows sharing of fear and anxiety; economic hardship; and other sensitive personal challenges to share them in the safe space that the internet permits. Moreover, non-physician peer leaders can be recruited from the same communities as the patients they serve, allowing increased trust.

## Policy-insurance reimbursement and coverage and increased funding for NCD treatment in LMICs

The COVID-19 pandemic has highlighted the importance of ensuring access to high quality care and the critical role of universal health coverage (UHC). Without additional measures such as changes in insurance reimbursement and coverage to improve access to treatment, limited resources and overwhelmed health systems will prevent countries from prioritizing the control of NCDs during this pandemic. This will likely lead to increased mortality due to COVID-19.

Most LMICs rely on private health expenditures from out-of-pocket payments and voluntary health insurance, resulting in high out of pocket expenditure and lack of access to the poor ^20^. Although the chronically ill in LMICs sometimes have greater access than healthier persons to health insurance, more can be done to ensure access to care for this population. Out-of-pocket costs should be waived for the duration of the emergency. 

Governments should consider strategies such as direct payments to providers and employ tools such as hazard pay, overtime pay, and rate increases to assure access to high quality care. In the long term, COVID-19 may prompt further study into healthcare finance models during emergencies. This research can advance health systems towards universal care models, allowing persons with NCDs to receive quality healthcare services that results in improved health outcomes.

## Prescription policies for drugs to treat NCDs

In order to minimize risk of exposure to SARS-CoV-2 in healthcare settings, prescription policies for the management of NCDs should be adjusted to minimize or eliminate requirements for face to face visits. For instance, medications to manage NCDs should be adjusted from 30- to 90-day prescriptions for stable patients. Medications should be delivered to communities to lower the risk of nosocomial transmission in healthcare settings where suspected cases may be receiving care. Fixed-dose combination therapy, also referred to as single pill combinations, is considered a best practice to improve adherence to treatment of NCDs. For this reason, the WHO added fixed-dose combination antihypertension medications to the Essential Medicines List in July 2019 ^21^. Thus, providers should consider adjusting medications to fixed dose combination pills to improve medication adherence and reduce patient burden.

## Community partnerships

Solving the complex health disparities impacting vulnerable populations requires the collaboration of diverse stakeholders within healthcare, public health, social services, and government. These partnerships must leverage community-based participatory research (CBPR) and relationship-centered principles to enhance the innovation, relevance, and effectiveness of interventions, programs, and policies ^22^. CBPR principles include building upon resources within the community; facilitating collaborative, equitable partnerships; focusing on local public health problems; and committing to long-term sustainability ^23^. Relationship-centered principles include respect for diverse perspectives, clear communication, sharing of power and partnership in decision-making ^24^. Such partnerships provide safe spaces for participants to examine how institutional, and interpersonal discrimination might impede their success, and to dismantle the impact of these structural and interpersonal behaviors on vulnerable groups ^25^.

## Conclusion

The COVID-19 pandemic has highlighted the fragility of healthcare systems and existing inequities in access to care and health outcomes for vulnerable populations across the globe. These inequities are particularly acute for the care of NCDs, which in turn impact outcomes for COVID-19. Additional research is needed to examine the impact of COVID-19 on the provision of NCD services, as well as treatment parameters. The pandemic response is a unique opportunity to shore up our approach to NCD care and pandemic planning worldwide, by employing innovative strategies that mitigate COVID-19 care disruptions and address the specific needs of these patients and communities in a sustainable fashion. Policy solutions that leverage robust prior evidence – and adapt these findings to the unique local threats created by COVID-19 – should prioritize the health and well-being of the most vulnerable, ensuring that no one is left behind during the COVID-19 response.

## Data availability

### Underlying data

No data are associated with this article.
