# Supplementary material for: Protecting the vulnerable during COVID-19: Treating and preventing chronic disease disparities
Source: Gates Open Res. 2020 Sep 9;4:125. [Version 1] doi: 10.12688/gatesopenres.13181.1 (PMC7578408; doi:10.12688/gatesopenres.13181.1)
Supplement: Peer review history

# Peer review: Reviewer response for version 1

**DOI:** 10.21956/gatesopenres.14385.r29739

**Published:** 2020-10-20

**Authors:** Tina Q. Tan

**Affiliations:** 1 Feinberg School of Medicine, Northwestern University, Chicago, IL, USA

Mobula and colleagues wrote a very concise open letter entitled "Protecting the vulnerable during COVID-19: Treating and preventing chronic disease disparities". The following are comments and questions regarding the article. The COVID-19 pandemic brought to the forefront and further accentuated the health disparities and increased rates of chronic non-communicable diseases (NCDs) in vulnerable populations that have existed for long periods of time pre-COVID-19. The pandemic not only served as a catalyst to put a spotlight on the increased risk for severe COVID-19 disease, hospitalization and death in these populations, but also exposed the long standing health disparities suffered by these individuals and the contribution of these disparities to severe COVID-19 disease. 

 The article is very well written and provides proposed strategies to address protecting these vulnerable communities from NCDs.

1. Telehealth, M-health and other technologies strategy - even though 70% of mobile subscriptions are in LMICs, do these subscriptions have the bandwidth and data needs to support a telehealth app?
2. Shifting and sharing health tasks across the healthcare team strategy:
  a. The authors may want to reword the first sentence since some of the task-shifting examples are not primarily physicians' tasks and are already being conducted by other non-physician healthcare providers.
3. Policy-insurance reimbursement and coverage strategy:
  a. This is not just a problem in LMICs but is also a problem here in the US. A large portion of the vulnerable populations have no insurance or are underinsured.

Does the article adequately reference differing views and opinions?

Yes

Are all factual statements correct, and are statements and arguments made adequately supported by citations?

Yes

Is the Open Letter written in accessible language?

Yes

Where applicable, are recommendations and next steps explained clearly for others to follow?

Yes

Is the rationale for the Open Letter provided in sufficient detail?

Yes

Reviewer Expertise:

pneumococcal epidemiology and PCV impact, global pertussis epidemiology and impact of pertussis containing vaccines, vaccine education for healthcare providers and patients

I confirm that I have read this submission and believe that I have an appropriate level of expertise to confirm that it is of an acceptable scientific standard.

# Peer review: Reviewer response for version 1

**DOI:** 10.21956/gatesopenres.14385.r29740

**Published:** 2020-10-12

**Authors:** David M. Mannino

**Affiliations:** 1 Department of Preventive Medicine and Environmental Health, College of Public Health, University of Kentucky, Lexington, KY, USA

- This is a nice overview of providing care for non communicable diseases in the setting of Covid-19, which has disrupted the usual care pathways.
- The letter is well written and easy to follow.
- If a figure or table is allowed - this may be useful to add to summarize the 7 recommendations in a single place.
- Although much of the focus in the letter was LMIC's, all of these issues are important in high income countries (and this should be emphasized).

Does the article adequately reference differing views and opinions?

Yes

Are all factual statements correct, and are statements and arguments made adequately supported by citations?

Yes

Is the Open Letter written in accessible language?

Yes

Where applicable, are recommendations and next steps explained clearly for others to follow?

Yes

Is the rationale for the Open Letter provided in sufficient detail?

Yes

Reviewer Expertise:

COPD

I confirm that I have read this submission and believe that I have an appropriate level of expertise to confirm that it is of an acceptable scientific standard.
